# Non-invasive plasma testing for *CD274* UTR structural variations by next-generation sequencing in cancer

**DOI:** 10.1038/s41420-023-01316-1

**Published:** 2023-01-30

**Authors:** Wenjuan Zhang, Jian Cao, Ke Liu, Ziwei Qu, Ying Zheng, Jun Yu, Yishan Yu, Yongsheng Wang, Wendy Wu

**Affiliations:** 1 Berry Oncology Corporation, Beijing, China; 2 Department of Radiation Oncology, Shandong Cancer Hospital and Institute, Shandong First Medical University and Shandong Academy of Medical Sciences, Jinan, China; 3 Thoracic Oncology Ward, Cancer Center, West China Hospital, Sichuan University, Chengdu, China

**Keywords:** Diagnostic markers, Tumour biomarkers

## Abstract

Immunotherapy is now the main choice of systemic therapy for many cancer patients, while current biomarkers for tumor immunotherapy may be limited by the accessibility of patient tumor tissue or tumor neoplastic content. Rare mutation in the 5’ and 3’-untranslated region (UTR) of *CD274* gene (Protein name: PD-L1) has been recently reported in hematologic and solid tumors as a potential biomarker for assessing efficacy during immunotherapy. However, multi-omics analysis for *CD274* UTR region, especially circulating tumor DNA (ctDNA), have been little explored in the pan-cancer perspective. We developed a cSMART2.0 technology featured with higher capture efficiency and homogeneity to detect this rare structural variant in 2249 Chinese patients’ cohort with multiple cancers. An incidence of 0.36% was detected in this cohort, consistent with TCGA (The Cancer Genome Atlas), while the prevalence of SV in *CD274* UTR region in liver and breast cancer were significantly higher than TCGA. The liquid biopsy result from ctDNA was 100% concordance with gDNA result getting from tumor tissue detection, and further validated by immunohistochemistry (IHC) and multiplex immunofluorescence (mIF) experiments. Patients carrying this SV in *CD274* UTR region without driver gene mutation responded to immune checkpoint inhibitors (ICIs). This study proves that rare structural variants in *CD274* UTR region exist in various cancer in Chinese population for the first time, which can induce immune escape and be used for prediction of response to ICIs. Liquid biopsy based cSMART 2.0 technology could offer more sensitive and accurate detection to navigate potential ICIs patients and to benefit patients with advanced disease when tissue samples are not available.

## Introduction

Immune checkpoint inhibitors (ICIs) targeting PD-L1 have become the primary choice of systemic therapy for most cancer patients [1]. Current biomarkers for tumor immunotherapy mainly include co-positive score (CPS) for PD-L1 expression, microsatellite instability/defective mismatch repair (MSI/dMMR) status and tumor mutation burden (TMB) [2]. However, these biomarkers are mostly limited by the accessibility of patient tumor tissue or tumor neoplastic content, and patients screened by these biomarkers have a high indeterminacy of response [3].

Peripheral blood-based detection has the advantage of easy sampling and comprehensive information from both tumor and patient immune status, and immunotherapy-related biomarkers getting from liquid biopsy become valuable in predicting the efficacy of several immunotherapies and monitoring the dynamics of treatment response [4]. While the consistency of peripheral blood biomarker assays with tissue assays is controversial due to limitations in assay technology and analytical methods [5]. There is an urgent need to develop more accurate and sensitive circulating tumor DNA (ctDNA) assays to navigate the patients potentially responsive to immunotherapy.

Kataoka’s study showed that rare structural variants in the UTR region (including 5' UTR and 3' UTR) of *CD274* (Protein name: PD-L1) gene could increase the expression level of PD-L1 protein in a variety of tumors, which might induce the development of immune escape behavior in tumor cells [6]. Previous studies on molecular mechanisms have revealed that mutations in the UTR region of *CD274* gene can instigate the immune escape of tumor cells by inhibiting the microRNA suppressive regulation of PD-L1 expression [7]. Mutation in the 3' UTR region of *CD274* gene has been recently reported in hematologic [8] and solid tumors [9] as a potential biomarker for assessing efficacy during immunotherapy, suggesting the rare variants in the UTR region of *CD274* gene to guide tumor immunotherapy. To our best knowledge, multi-omics analysis of *CD274* UTR regions, especially at the ctDNA level, has been little investigated in a pan-cancer manner.

Here, we developed a liquid biopsy-based cSMART2.0 technology (Supplementary Fig. 1, Supplementary Table 1), which utilizes a modified probe capture method for the enrichment of target genes and allows a highly sensitive and specific detection of multiple mutation types in multiple genes via high-throughput sequencing based on high capture efficiency and homogeneity. cSMART 2.0 (Supplementary Fig. 1) was used to detect rare variants in the UTR region of *CD274* gene in multi-omics methodology for the first time in a large-scale retrospective cohort covered a variety of tumor types, while correlating their presence with the efficacy of immunotherapy.

## Results

### Frequency of *CD274* UTR region variants

**Fig. 1:**
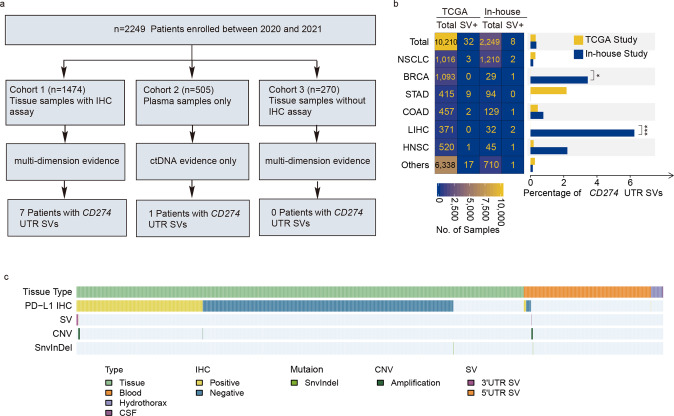
Study design and landscape of CD274 UTR variants. **a** Study design. **b** The heatmap showes the number of total patients and patients with CD274 UTR SVs in TCGA study and our study, respectively. The barplot showes the proportion of CD274 UTR SVs in TCGA study and our study, respectively. *P*-values were calculated by fisher’ exact test, *p < 0.05, **p < 0.01, ***p < 0.001. NSCLC non-small cell lung cancer, BRCA breast cancer, GC gastric cancer, CRC colorectal cancer, LC liver cancer, HNSC head-neck squamous cell carcinoma. **c** Genomic landscape of CD274 variants in pan-cancer.

In the present study, we enrolled 2249 patients diagnosed with various tumor types between August 2017 and February 2021 (Fig. 1a). Among 2249 patients, 1474 patients can obtain tissue and plasma samples at the same time, as well as immunohistochemistry (IHC) test results of tissue samples, named as “tissue samples with IHC assay results” group and performed multi-omics assays; 270 patients could obtain tissue and plasma samples at the same time, but lack of IHC test results, named as “tissue samples without IHC assay results” group; 505 patients only have plasma samples and just performed ctDNA detection.

We identified structural variants in *CD274* UTR region with an incidence of 0.36% in pan-cancer, consistent with the incidence of 0.31% in TCGA (The Cancer Genome Atlas) [6], a landmark cancer genomics program, molecularly characterized 33 cancer types through large-scale genome sequencing and comprehensive multidimensional analysis. But the incidence of liver and breast cancer in our cohort, 6.25% and 3.44%, were significantly higher than TCGA, not detected (Fig. 1b). As to the variation types of *CD274* gene, the most frequent genomic alteration in the UTR region were structural variation (SV) and copy number variation (CNV), neither single nucleotide variants (SNV) nor indels (Fig. 1c).

### Multi-omics analysis on the rare variants of CD274 UTR region

**Fig. 2:**
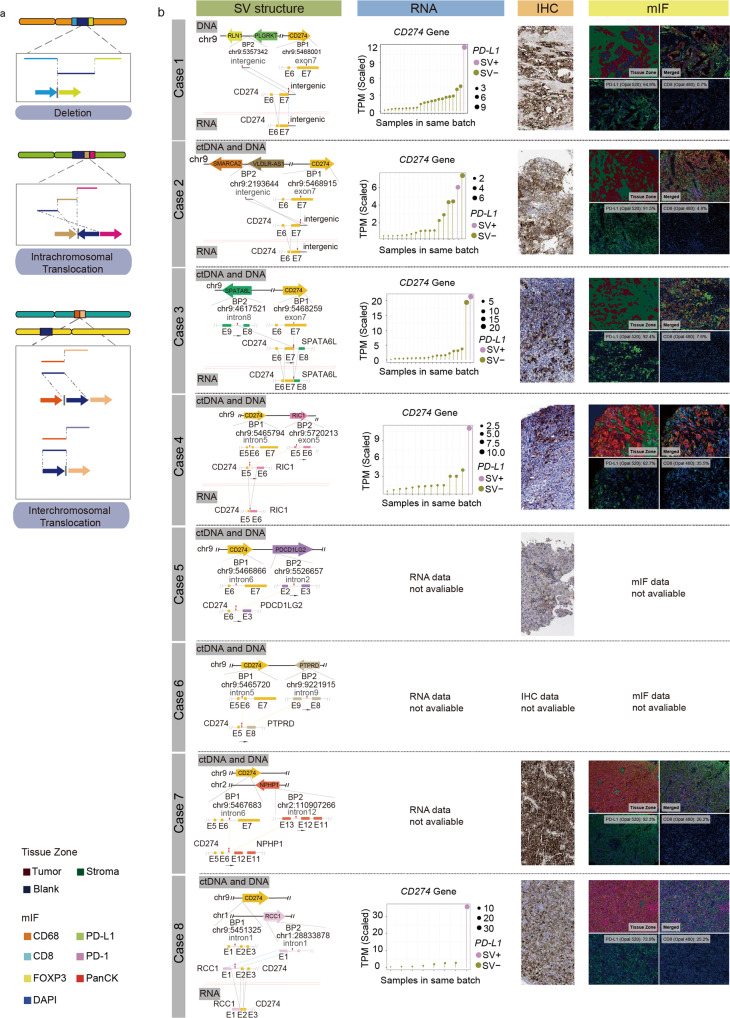
Classification and multi-omics analysis on the SV of CD274 UTR region. **a** Classification of structural variants in CD274 UTR region. **b** From left to right, the first column represents the SV structure of CD274 UTR SVs at DNA and RNA levels; the second column represents the CD274 mRNA expression of patients with CD274 UTR SV comparing to samples in the same batch; the third column represents the PD-L1 IHC results; the fourth column represents the mIF results.

**Fig. 3:**
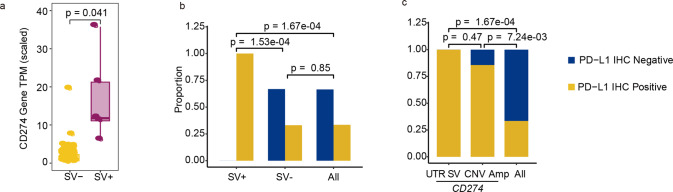
Correspondence of SV of CD274 UTR region detected by tissues and those detected by ctDNA. **a** Boxplot showing the CD274 mRNA expression between patients with CD274 UTR SV (SV+) and patients without CD274 UTR SVs (SV-). *P*-values were computed using Wilcoxon test. **b** Barplot showing the proportion of patients with PD-L1 IHC positive among patients with CD274 UTR SV (SV+), without CD274 UTR SV (SV-) and overall patients. *P*-values were calculated by fisher’ exact test. **c** Barplot showing the proportion of patients with PD-L1 IHC positive among patients with CD274 UTR SV, with CD274 CNV amplification and overall patients. *P*-values were calculated by fisher’ exact test.

We use cSMART 2.0 technology to comprehensively analyze the SV of both 3' UTR and 5' UTR and defined these variants as three types, intrachromosomal translocations, chromosomal deletions, and interchromosomal translocations (Fig. 2a). The multi-omics analysis results of all cases with this rare variant were shown as Fig. 2b, included 3 cases of intrachromosomal translocations (Cases 1, 2 and 3), 3 cases of chromosomal deletions (Cases 4, 5 and 6), and 2 cases of interchromosomal translocations (Cases 7 and 8). The ctDNA result showed 100% concordance with gDNA data in breakpoint position and structure detection, which was validated at the transcriptomics level (Fig. 2b, Table 1, Supplementary Fig. 2). RNA results showed that the breakpoint locations of DNA structural variants in *CD274* UTR region were recapitulated in the RNA structure detection. The scaled *CD274* gene TPM (Transcripts Per Million) in samples containing structural variants of the UTR region far exceeded those of other samples in the same batch (*P* = 0.043) (Fig. 3a). It suggests that structural variants in *CD274* UTR region would cause significantly higher expression at the transcriptional level. And all cases carrying structural variants in *CD274* UTR region have PD-L1 expression level >50% (Fig. 2b, Table 1).

**Table 1:** Clinical information of patients and mutations in *CD274* UTR region.

Case	Diagnosis	SV mut	Break Point Loc (ctDNA)	Break Point Loc (DNA)	Break Point Loc (RNA)	PD-L1 TPS	PD-L1 CPS	Content of tumor cells	*CD274* gene TPM
1	Non-Small Cell Lung Cancer	*CD274*- > intergenic[*RLN1*,*PLGRKT*]	–	inside[CD274:exon_7 | utr3;intergenic[RLN1,PLGRKT]]	inside[CD274:exon_7 | utr3;intergenic[RLN1,PLGRKT]]	95%	95	55%	6237.48
2	Nasopharyngeal Carcinoma	*CD274*- > intergenic[*SMARCA2*,*VLDLR-AS1*]	inside[*CD274*:exon_7 | utr3;intergenic[*SMARCA2*,*VLDLR-AS1*]]	inside[CD274:exon_7 | utr3;intergenic[SMARCA2,VLDLR-AS1]]	inside[CD274:exon_7 | utr3;intergenic[SMARCA2,VLDLR-AS1]]	90%	95	90%	5205.902
3	Intrahepatic Cholangiocarcinoma	*CD274*- > *SPATA6L*	inside[*CD274*:exon_7 | utr3;*SPATA6L*:intron_8]	inside[CD274:exon_7 | utr3;SPATA6L:intron_8]	inside[CD274:exon_7 | utr3;SPATA6L:exon_8]	60%	65	90%	19182.757
4	Liver Cancer	*CD274*- > *RIC1*	inside[CD274:intron_5;RIC1:exon_5]	inside[CD274:intron_5;RIC1:exon_5]	inside[CD274:exon_5;RIC1:exon_6]	60%	65	30%	11039.696
5	Non-Small Cell Lung Cancer	*CD274*- > *PDCD1LG2*	inside[CD274:intron_6;PDCD1LG2:intron_2]	inside[CD274:intron_6;PDCD1LG2:intron_2]	–	5%	15	60%	2612.93
6	Breast Cancer	*CD274*- > *PTPRD*	inside[CD274:intron_5;PTPRD:intron_9]	–	–	–	–	–	–
7	Colorectal Cancer	*CD274*- > *NPHP1*	inside[CD274:intron_6;NPHP1:intron_12]	inside[CD274:intron_6;NPHP1:intron_12]	–	90%	90	85%	–
8	T cell lymphoma	*SNHG3*- > *CD274*	inside[SNHG3:intron_1 | utr5;CD274:exon_2 | utr5]	inside[SNHG3:intron_1 | utr5;CD274:exon_2 | utr5]	inside[SNHG3:intron_1 | utr5;CD274:exon_2 | utr5]	30%	30	95%	28963.900

We divided 1474 patients of “tissue samples with IHC assay results” group into two subgroups as SV-positive and SV-negative. The proportion of patients with positive PD-L1 expression in SV-positive cohort (8/8) was significantly higher than SV-negative cohort (487/1467) (*P* = 1.53e-04) and overall patients (494/1474) (*P* = 1.67e-04) (Fig. 3b), which indicated that patients with positive PD-L1 expression were significantly enriched in the *CD274* UTR SV-positive group. Interestingly, PD-L1 positive samples were also enriched amongst patients carrying *CD274* gene copy number amplification (6/7), again significantly higher than the positive PD-L1 expression ratio in all patients (494/1474) (*P* = 7.24e-03) (Fig. 3c). However, there was no significant difference in the rate of positive PD-L1 expression in SV-positive versus CNV patients (*P* = 0.467) (Fig. 3c).

We investigated the role of SV in *CD274* UTR region in regulating the immune microenvironment of tumors and the immune escape behavior of tumor cells from a cytological perspective using multiplex immunofluorescence (mIF). A significant enrichment of PD-L1 positive cells in the tumor cell region were found in the SV positive patients (Fig. 2b). The ratio of the number of PD-L1 positive cells to the number of CD8 positive cells and the ratio of the number of PD-L1 positive cells to the number of CD68 positive cells were both >1 (Supplementary Table 2). The mIF results suggest that SV in *CD274* could promote the immune escape behavior of tumor cells.

### Presence of *CD274* UTR structural variants detected in ctDNA may predict benefit from immunotherapy

Mutations in *CD274* 3' UTR region have recently been reported as potential biomarkers for assessing the efficacy of immunotherapy in tumors [9]. We selected four typical patients to explore their clinical outcome of immunotherapy.

**Fig. 4:**
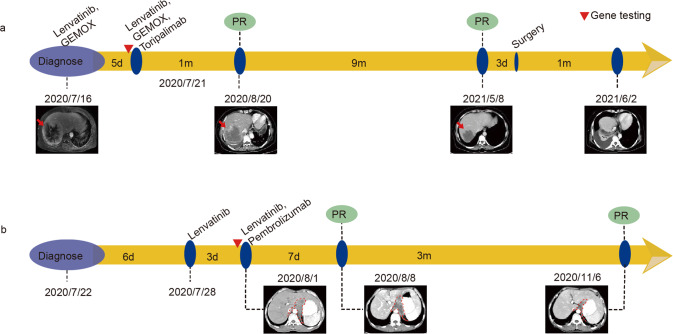
Timeline of disease, treatment history and CT scan images of patients. **a** Timeline of Case 3. **b** Timeline of Case 4. Radiological assessments were performed by RECIST (Response Evaluation Criteria in Solid Tumors) v1.1. Gene testing was marked by red inverted triangle.

Case 3 is an adult patient with intrahepatic cholangiocarcinoma (stage IVB, T2N1M1) with positive PD-L1 expression and disruption of the *CD274* 3' UTR region. Based on the genetic testing result, the treatment regimen was adjusted to chemotherapy and targeted drugs in combination with ICI, treprolizumab. The patient achieved partial response after treatment and was subsequently evaluated as suitable for surgical resection (Fig. 4a).

Case 4 is an adult patient with metastatic hepatocellular carcinoma (stage IVB) with positive PD-L1 expression and disruption of the *CD274* 3' UTR region. The patient’s treatment regimen was subsequently adjusted to lenvatinib plus ICI, pembrolizumab. The patient achieved partial response after treatment (Fig. 4b).

Case 6 is an adult patient with right-sided breast cancer with positive PD-L1 expression and disruption of the *CD274* 3' UTR region accompanied by driver gene *ERBB2* amplification. The patient’s treatment regimen was adjusted to ICI, sindilizumab, plus paclitaxel albumin. The patient unfortunately did not respond and passed away after one treatment cycle. The clinical outcome of Case 6 is consistent with the mechanism that driver gene mutations, such as *EGFR* and *c-MET*, upregulate PD-L1 protein expression and administration of ICI is less effective [10].

Case 8 is an adult patient with mature T-cell lymphoma with positive PD-L1 expression and fusion in the *CD274* 5' UTR region. Treatment for this patient was chemotherapy and adjusted to a PI3K inhibitor later. Unfortunately, the patient responded poorly and subsequently died.

## Discussion

In this study, we developed a cSMART2.0 technology featured by probe design and single-molecule capture method combining with precise SV analysis algorithm, which offers DNA and RNA detection with high sensitivity and specificity in low frequency mutations of multiple clinical scenarios, especially blood samples. Rare mutation in the 5'and 3' UTR region of *CD274* gene has been reported as a potential biomarker for assessing efficacy of immunotherapy, and cSMART2.0 was used to detect this rare mutation and identify somatic variation.

We demonstrated the detection rate of SV in *CD274* UTR region in a large-scale retrospective Chinese cohort for the first time, and found that the incidence ratio of SV in *CD274* UTR region was significantly higher than TCGA cohort in liver and breast cancer but consistent in pan-cancer. We speculated that the clinical significance of SV mutations in the UTR region of the *CD274* gene has long been less studied might be attribute to the complicated variation pattern and the consequent detection barrier, as well as their low prevalence. It requires SV-specific probe coverage and complex bioinformatics analysis strategies, especially higher technical sensitivity in blood samples to remove clonal hematopoiesis. cSMART 2.0 technology is invented to detect structure variation as accurate as SNV and CNV, more conventional mutation types, with its effective noise reduction, strong sensitivity and high coverage, which enhances the detect technique for this rare mutation (Supplementary Fig. 3).

Current *CD274* mutation detection usually needs tumor tissues from patients, which limits concomitant diagnosis and precise drug guidance for patients with no access to tumor tissue [11]. In previous studies, it remains controversial whether blood-based *CD274* assay results can reflect mutations or PD-L1 expression difference in-situ tumor tissue due to blood CTC-based PD-L1 assay relies on ELISA and qPCR techniques [12]. With cSMART 2.0 technology, we obtained 100% concordance between blood-based ctDNA and tumor tissue-based gDNA for *CD274* structural variation detection. Subsequently, we found that patients with structural variants in *CD274* UTR region who responded to immune checkpoint inhibitors were all positive for PD-L1 expression validated by mIF method, which confirmed the immune escape behavior of tumor cells. While patient carrying this rare variation and driver gene mutations did not respond to ICIs.

**Fig. 5:**
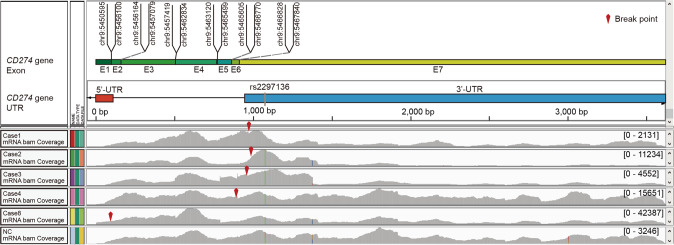
Molecular mechanisms of SV in the UTR region of CD274 gene induce immune escape. Visualization of RNA sequencing reads coverage in CD274 exon regions by the Integrative Genomic Viewer (IGV) software. Top panel (CD274 gene Exon) represents the relative length of each exon of CD274; second panel (CD274 gene UTR) marked the length and position of 5' UTR and 3' UTR of CD274; bottom panel represents mRNA bam coverage of five patients with CD274 UTR SV and a control sample without CD274 UTR SV.

Previous studies [13, 14] showed that SNV in the 3' UTR region of *CD274* gene is related to the binding of microRNAs and cause PD-L1 overexpression, which contributes to tumor cell immune escape. In this study, SV in 3' UTR region all occurred before the reported miRNA binding sites identified in the above studies, resulting in the inactivation of microRNA negative regulation on *CD274* gene expression and a significant increase of PD-L1 protein (Fig. 5). The molecular mechanism of SV in *CD274* 5' UTR region remains unclear till now, and in vitro studies revealed the presence of a binding site for the regulator eEF2K [15] and eIF5B [16] in this region and might trigger upregulation of PD-L1 expression. In our investigation, SV in *CD274* 5' UTR region induced a strong promoter of *RCC1* gene rearrange to *CD274* gene (Fig. 5, Fig. 2b), resulting in a significant increase in the expression of PD-L1 protein. Therefore, we speculate that the damage caused by SV in *CD274* UTR region is much more severe than that caused by SNP (Fig. 3a, b), and structural variation in *CD274* UTR region might induce two post-transcriptional regulation molecular mechanism, one is that the higher efficient promoter fusing with the 5' UTR region and activating mRNA transcription; the other is that the miRNA binding site in the 3' UTR region is deleted, both leading to increased expression of PD-L1 protein and immune escape behavior of tumor cells (Supplementary Fig. 4).

Our study still has some limitations. First, an expanded prospective cohort study is need to validate the relationship between structural variants in the UTR region of the *CD274* gene and the efficacy of ICI therapy in this study. Second, the frequency of structural variants in *CD274* UTR region in patients with more types of tumors and the relationship with the efficacy of immune checkpoint inhibitor therapy also need to be further investigated. Third, the molecular mechanism of expression changes caused by SV in the UTR region of the *CD274* gene, which leads to immune escape of tumor cells and may serve as a biomarker of ICI efficacy, requires further molecular cell biology experiments to confirm.

In summary, we demonstrated the incidence of structural variation in *CD274* UTR region in a large-scale retrospective Chinese cohort for the first time. The liquid biopsy result from ctDNA was 100% concordance with gDNA result getting from tumor tissue detection, and further validated by IHC and mIF experiments. With its more sensitive and accurate detection performance, cSMART2.0 technology offers more solutions for improving the detection dilemma mainly limited by experimental methods and analysis strategies, navigating the potential ICI beneficial patients, and assisting patients with advanced disease when tissue samples are not available.

## Supplementary information

Additional File 1

Additional File 2

Additional File 3

Additional File 4

Additional File 5

Additional File 6

Additional File 7

Additional File 8

## Data Availability

The data are available from the corresponding author on reasonable request.
