# Supplementary material for: Non-invasive plasma testing for CD274 UTR structural variations by next-generation sequencing in cancer
Source: Cell Death Discov. 2023 Jan 30;9:35. doi: 10.1038/s41420-023-01316-1 (PMC9887064; doi:10.1038/s41420-023-01316-1)
Supplement: Supplementary file 1 — Additional File 1 [file 41420_2023_1316_MOESM1_ESM.docx]

**Additional File 1**

**Supplementary Materials and Methods**

***Patients***

Samples from a cohort of 2249 patients with tumor from August, 2020, to February, 2021 were collected for multi-omics assays. Among all patients, 1474 patients in Cohort 1 can obtain tissue samples and plasma samples at the same time, and IHC test results could be obtained from tissue samples; 270 patients in Cohort 2 could obtain tissue samples and plasma samples at the same time, but IHC test results could not be obtained; 505 patients in Cohort 3 can only obtain plasma samples. The patients in Cohort 1 performed multi-omics assays, the patients in Cohort 2 performed multi-omics assays except IHC, while the patients in Cohort 3 only performed ctDNA assay. All patients signed the informed consent for the study (Supplementary Figure 2A).

***cSMART 2.0 technology***

cSMART 2.0 technology includes sample extraction, library construction, DNA sequencing and bioinformatics analysis (Supplementary Figure 1C). It is an independent technology and development program based on large Panel full exon region capture detection, and serves for somatic gene detection of patients with intermediate and advanced tumors. It can be applied to multiple clinical scenarios such as tissue, plasma, pleural fluid, cerebrospinal fluid and other sample types with detection of multiple omics including DNA, RNA and ctDNA. In particular, detecting low-frequency mutations in blood samples requires high technical sensitivity, which is difficult to be achieved by conventional mutation detection techniques. cSMART 2.0 technical principle: the Unique Molecular identifier Group (UMG) can mark the original fragments from the same sample with unique marker when constructing the library (Supplementary Figure 5A). The probe design and experimental scheme combined with the bioinformatics analysis scheme can more effectively analyze SNVs, indels, CNVs, SVs and TMB, and reduce the influence of systematic error and germ line background noise (Supplementary Figure 5B).

*Nucleic Acid Extraction*

Genomic DNA and total RNA were simultaneous purified from the formalin-fixed paraffin-embedded (FFPE) tumor samples using AllPrep DNA/RNA FFPE Kit (Qiagen, Hilden, Germany). Approximately 10 mL peripheral blood sample were collected using Cell-Free DNA BCT tube (Streck, Omaha, USA). Plasma and buffy coat were then separated from the peripheral blood according to manufacturer’s protocol. Cell-free DNA was extracted from the entire plasma sample by QIAamp Circulating Nucleic Acid Kit (Qiagen, Hilden, Germany). As a control, genomic DNA was extracted from 50 µL buffy coat by MagPure Tissue&Blood DNA LQ Kit (Magen, Guangzhou, China). Nucleic acid concentration was determined with the Qubit HS dsDNA kit or Qubit RNA HS Assay Kit (Invitrogen, Carlsbad, USA).

*NGS Library Construction and Sequencing*

Cancer mutation profiling of the samples was performed using Solid Tumor Comprehensive Test (STCT) or Blood Cancer Comprehensive Test (BCCT) (provided by Berry Oncology Co., Ltd., Fuzhou, China). The STCT includes a 654-gene DNA assay and a 105-gene RNA assay. The BCCT includes a 629-gene DNA assay and a 105-gene RNA assay. The DNA assay detects somatic mutations including SNVs, indels, CNVs and rearrangements at DNA level in FFPE or plasma samples, and uses WBCs as paired normal samples. The RNA assay detects rearrangements and splicing mutations at RNA level in FFPE samples. The 654-gene panel targets the entire coding sequence, intron 5 - intron 6 and 3’ UTR of CD274. The 629-gene panel targets the entire coding sequence and intron 1 of CD274. The 105-gene panel targets all exons of CD274. Methods of NGS library construction are described as below.

DNA library was constructed using CS2.0 Tissue DNA Library Prep Kit and CS2.0 cfDNA Library Prep Kit (Berry Oncology, Fujian, China). In brief, fragmented gDNA and cfDNA were end-repaired and A-tailed, followed by ligation with adapters containing UMG (Supplementary Figure 1C). Ligation products were purified and amplified by PCR. RNA library was constructed by CS2.0 RNA Library Prep Kit (Berry Oncology, Fujian, China). DNA (Supplementary Figure1B) and RNA (Supplementary Figure 1A) libraries were enriched for the targeted genes regions by liquid hybridization using CS2.0 DNA Hybridization and Wash Kit (Berry Oncology, Fujian, China). The enriched library is quantified by real-time PCR and sequenced using NovaSeq 6000 (Illumina, San Diego, USA) in pair-end 150 bp mode.

*NGS Quality Control*

Samples that fulfilled the following QC criteria were eligible for subsequent bioinformatics analysis. The specific criteria are as follows: average depth of the target area, leukocyte samples >200X, plasma samples >15000X, and tissue samples >1500X are required, and the overall coverage ratio of the target area is 97% or more, with homogeneity >80% and Q20 >85%.

*Bioinformatics*

BerryOncology™ MutLoc algorithm is a SNV/Indel detection tool for NGS combines tissue or plasma samples with WBC controls to effectively remove germline polymorphic mutations, clonal hematopoiesis, and systematic errors. The performance of low-frequency mutation detection is further improved by UMG probes. In addition, specific SNP polymorphism sites can further eliminate heterologous DNA interference (Supplementary Figure 5A). BerryOncology™ structural variation analysis tool was analyzed split-reads and Discordant Read Pair (DRP) signals to identify large fragment deletion or rearrangement and other structural variations in DNA and RNA samples (Supplementary Figure 5B). BerryOncology™ copy number detection algorithm includes CLD (Corrected Log2 Depth) and PoN (Pool of Normal) methods, in which normal human white blood cells are used to construct the background pool of tumor tissue sample, and normal human cfDNA are used to construct a background pool of plasma sample.

***IHC***

Four-µm FFPE slides of each case were evaluated for PD-L1 expression by immunohistochemistry. The experiment was performed using the VENTANA PD-L1 (SP263) Assay (Ventana Medical Systems, Inc., Tucson, US) or PD-L1 IHC 22C3 pharmDx kit (Dako North America Inc., Carpinteria, US) according to the manufacturer's instructions.

***Multiplex immunohistofluorescence assay***

Multiplex immunohistofluorescence was performed by using the following antibodies: CD8 (SP16; ZSGB-BIO), PD-1 (UMAB199; ZSGB-BIO), PD-L1 (SP263; Ventana), FoxP3 (236A/E7; Abcam), CD68 (PG-M1; ZSGB-BIO) and PanCK (AE1/AE3; ZSGB-BIO). Using an Opal 7-Color Automation IHC Kit (Akoya Biosciences), an optimized multiplex for CD8, PD-1, PD-L1, FoxP3, PanCK, CD68 and DAPI was conducted on a Leica Bond RXm fully automated immunostainer. Biomarkers that could co-localize in the same cellular compartment were paired with a spectrally separated Opal fluorophore to avoid potential spectral interference, as recommended by the manufacturer. The slides were scanned using the PerkinElmer Vectra Polaris system, which generates a single unmixed whole slide scan (Pixel resolution: 0.50 µm (20x)) of up to 7 colors. This single image equipped with a rapid application of Digital Image Analysis (DIA) could provide comprehensive information without stitching many spectrally unmixed image tiles. Stained slides were digitized using a multispectral slide-imaging platform (Phanochart, Akoya Biosciences). After tissue segmentation and digital cell phenotyping, the density and positive rate of each biomarker was semi-automatically assessed using the inForm Advanced Image Analysis software (inForm 2.5.1; Akoya Biosciences).

***Statisticis Analysis***

All statistical analyses are conducted using R script (v3.6). Two-sided Fisher’s exact test is utilized for analyzing the difference of CD274 UTR SV proportion between different cohorts and subgroups. Wilcoxon test is used to comparing CD274 differential mRNA expression between CD274 UTR SV-positive and CD274 UTR SV-negative patients. Statistical significance is defined as P value ≤ 0.05 (two-sides).
