# Supplementary material for: Non-invasive plasma testing for CD274 UTR structural variations by next-generation sequencing in cancer
Source: Cell Death Discov. 2023 Jan 30;9:35. doi: 10.1038/s41420-023-01316-1 (PMC9887064; doi:10.1038/s41420-023-01316-1)
Supplement: Supplementary file 2 — Additional File 2 [file 41420_2023_1316_MOESM2_ESM.docx]

Additional File 2

Figure Legends and Table Legends

Supplementary Figure 1.Experimental methodology.

a. RNA probe coverage strategy: when a gene has multiple transcripts, each transcript exon will be covered head and tail, and a master transcript will be selected for full coverage.

b. DNA probe coverage strategy: genes that often undergo structural variation, cover the exons and introns except for the repeat region.

c. cSMART 2.0 technical principle: the UMG can mark the original fragments from the same sample with unique marker when constructing the library.

**Supplementary Figure 2.Multi-omics results of patients with CD274 UTR SVs.**

a-h. From left to right, the first/second/third column represents the ctDNA, tissue DNA, RNA sequencing reads indicating CD274 UTR regions were visualized by the Integrative Genomic Viewer (IGV) software, respectively.

Case 1 (a), Case 2 (b) and Case 3 (c) are consistent with intrachromosomal translocation. A homologous sequence between the RLN1 and PLGRKT genes was inserted in the CD274 3' UTR region upstream of the homologous chromosome in genome of Case 1 (a); the CD274 3' UTR region Case 2 (b) was inserted with an inverted sequence located upstream of the homologous chromosome, between the SMARCA2 and VLDLR-AS1 genes; a sequence on the exon of the SPATA6L gene upstream of the homologous chromosome was inserted in the CD274 3' UTR region in the genome of Case 3 (c).

The patterns of mutation in Case 4 (d), Case 5 (e), and Case 6 (f) are consistent with chromosomal deletion. A deletion from intron 5 to exon 7 and 3'UTR of the CD274 gene was observed on chromosome 9 of Case 4 (d) and was replaced by the sequence of the RIC1 gene starting from exon 4. A deletion from intron 6 to exon 7 and 3'UTR of CD274 gene was observed on chromosome 9 in Case 5 (e) and was replaced by the sequence of PDCD1LG2 gene starting from intron 2. A deletion from intron 5 to exon 7 and 3'UTR of the CD274 gene was observed on chromosome 9 of Case 6 (f) and was replaced by the sequence of the PTPRD gene starting from intron 9.

The pattern of mutation in Case 7 (g) and Case 8 (h) is consistent with interchromosomal translocation. In case 7 (g), intron 6 of the CD274 gene on chromosome 9 and intron 12 of the NPHP1 gene on chromosome 2 were simultaneously interrupted, producing an interchromosomal balanced translocation with disruption of exon 7 of the CD274 gene as well as the 3' UTR. For Case 8 (h), Intron1 of the CD274 gene on chromosome 9 and Intron1 of the RCC1 gene on chromosome 1 were broken simultaneously, followed by gene fusion between the upstream sequence of the RCC1 gene breakpoint and the downstream sequence of the CD274 breakpoint, and finally the CD274 gene exon 1 and 5'UTR were replaced with the promoter region of the RCC1 gene.

Supplementary Figure 3.Methods of somatic mutation and SV calling.

A. In the process of mutation calling, the Unique Molecular identifier Group (UMG) tag is used to reduce the noise of NGS data to ensure the authenticity of mutations.

B. Split Reads and Discordant Read Pair signals are used to identify structural variation.

**Supplementary Figure 4. Molecular mechanisms of SV in the UTR region of CD274 gene induce immune escape of tumor cells and participate in tumor suppression by immune checkpoint inhibitors.**

**Supplementary Table 1. Concurrent alterations identified in patients.**

**Supplementary Table 2. mIF results.**
