# Supplementary figures and images for: Non-invasive plasma testing for CD274 UTR structural variations by next-generation sequencing in cancer
Source: Cell Death Discov. 2023 Jan 30;9:35. doi: 10.1038/s41420-023-01316-1 (PMC9887064; doi:10.1038/s41420-023-01316-1)

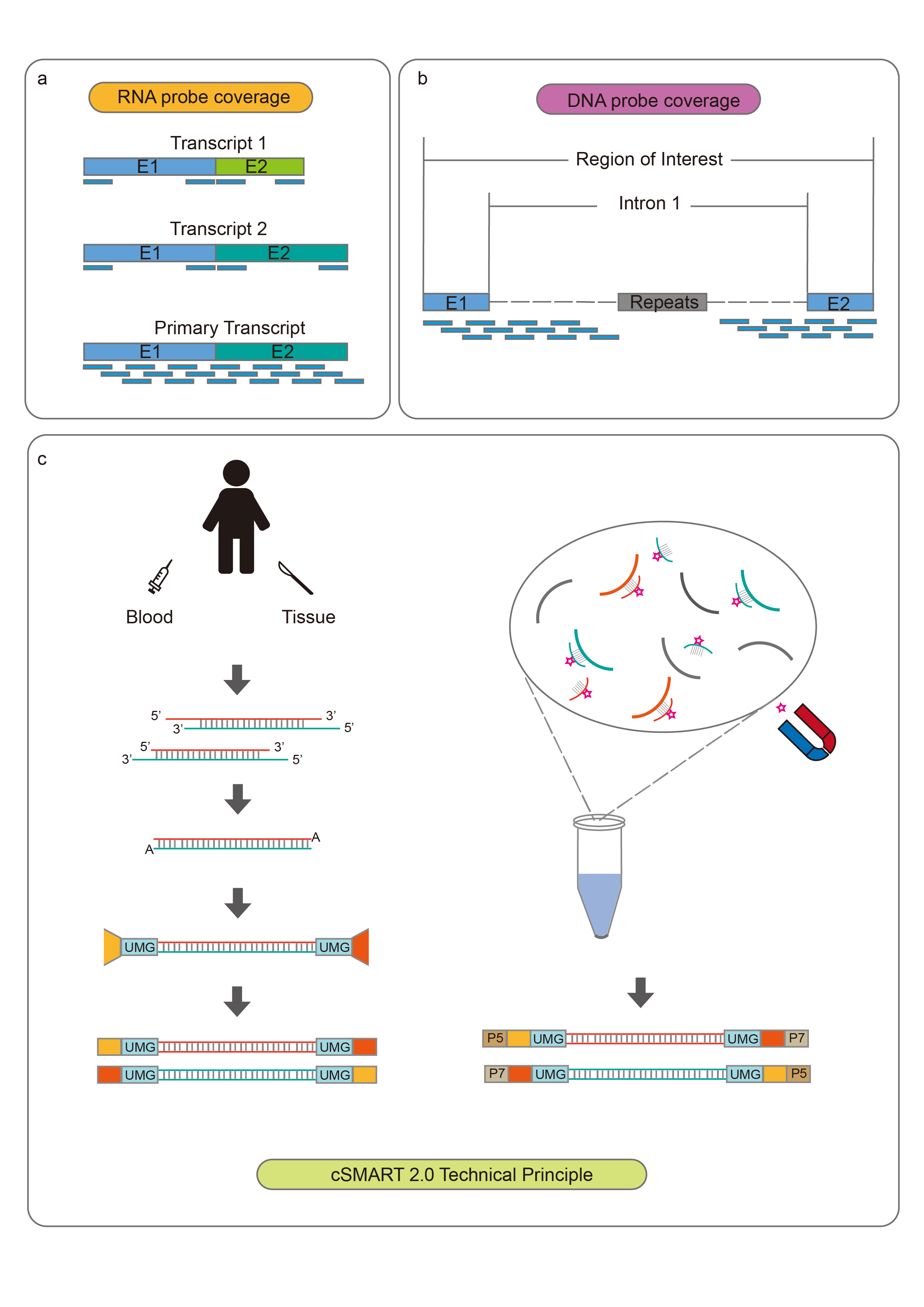

Supplement: Supplementary file 3 — Additional File 3 [file 41420_2023_1316_MOESM3_ESM.png]

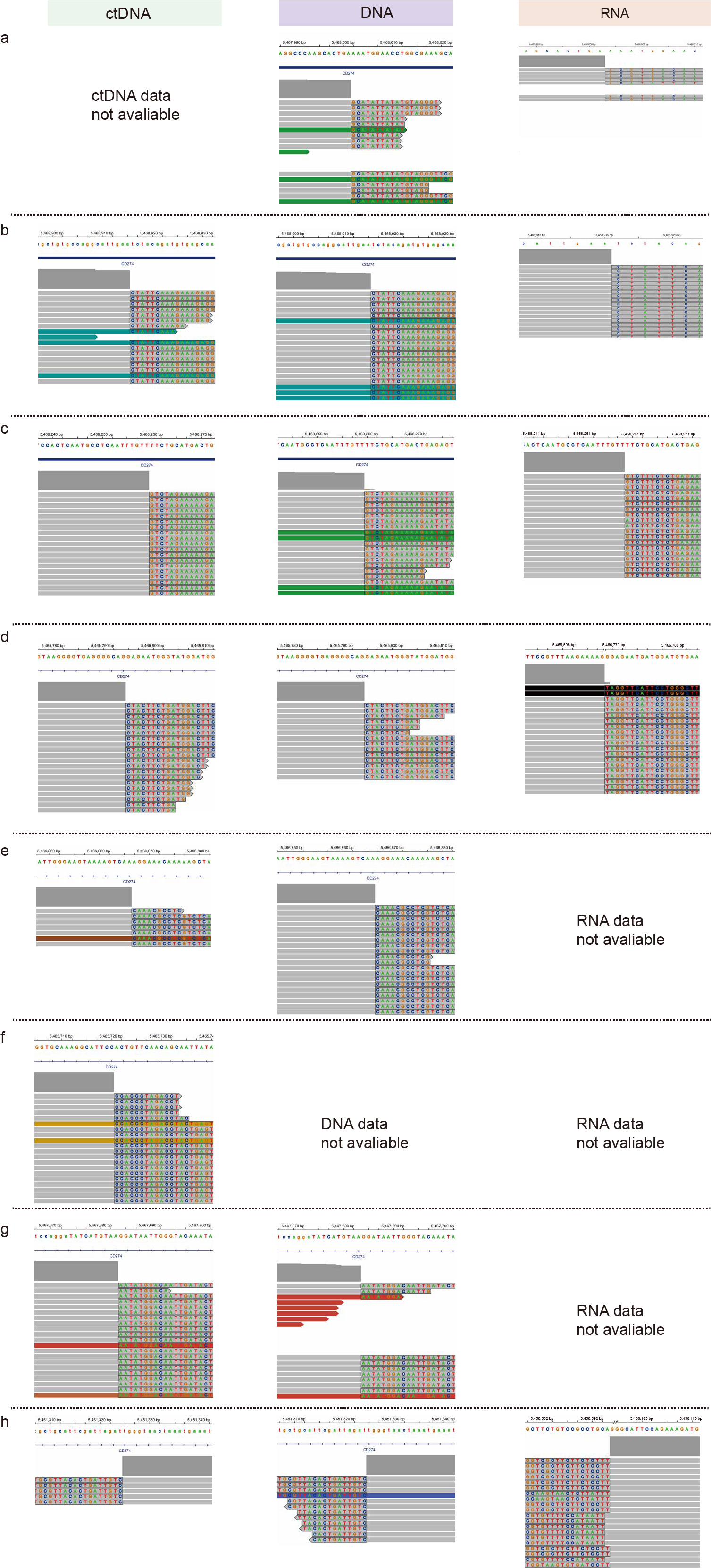

Supplement: Supplementary file 4 — Additional File 4 [file 41420_2023_1316_MOESM4_ESM.png]

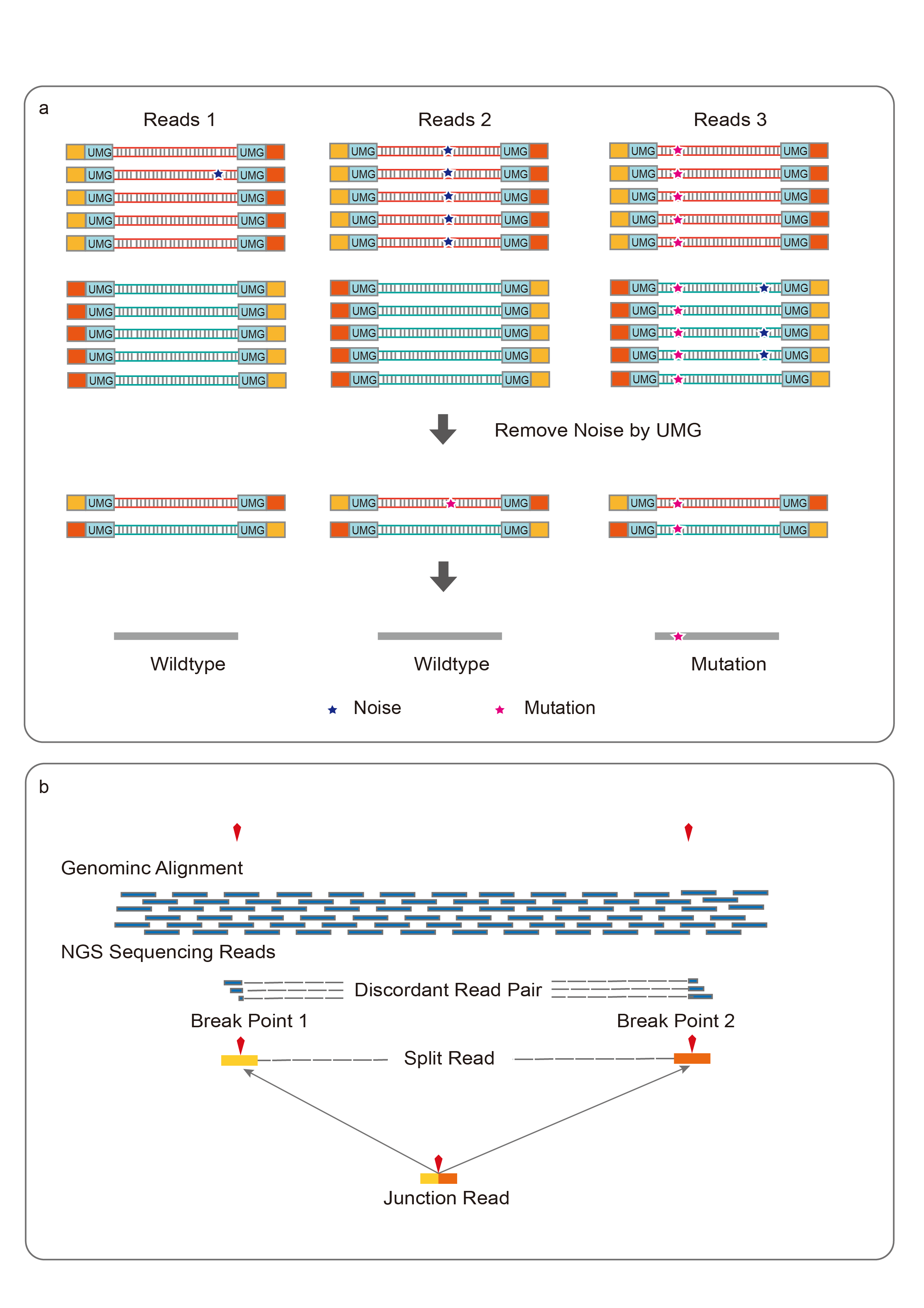

Supplement: Supplementary file 5 — Additional File 5 [file 41420_2023_1316_MOESM5_ESM.png]

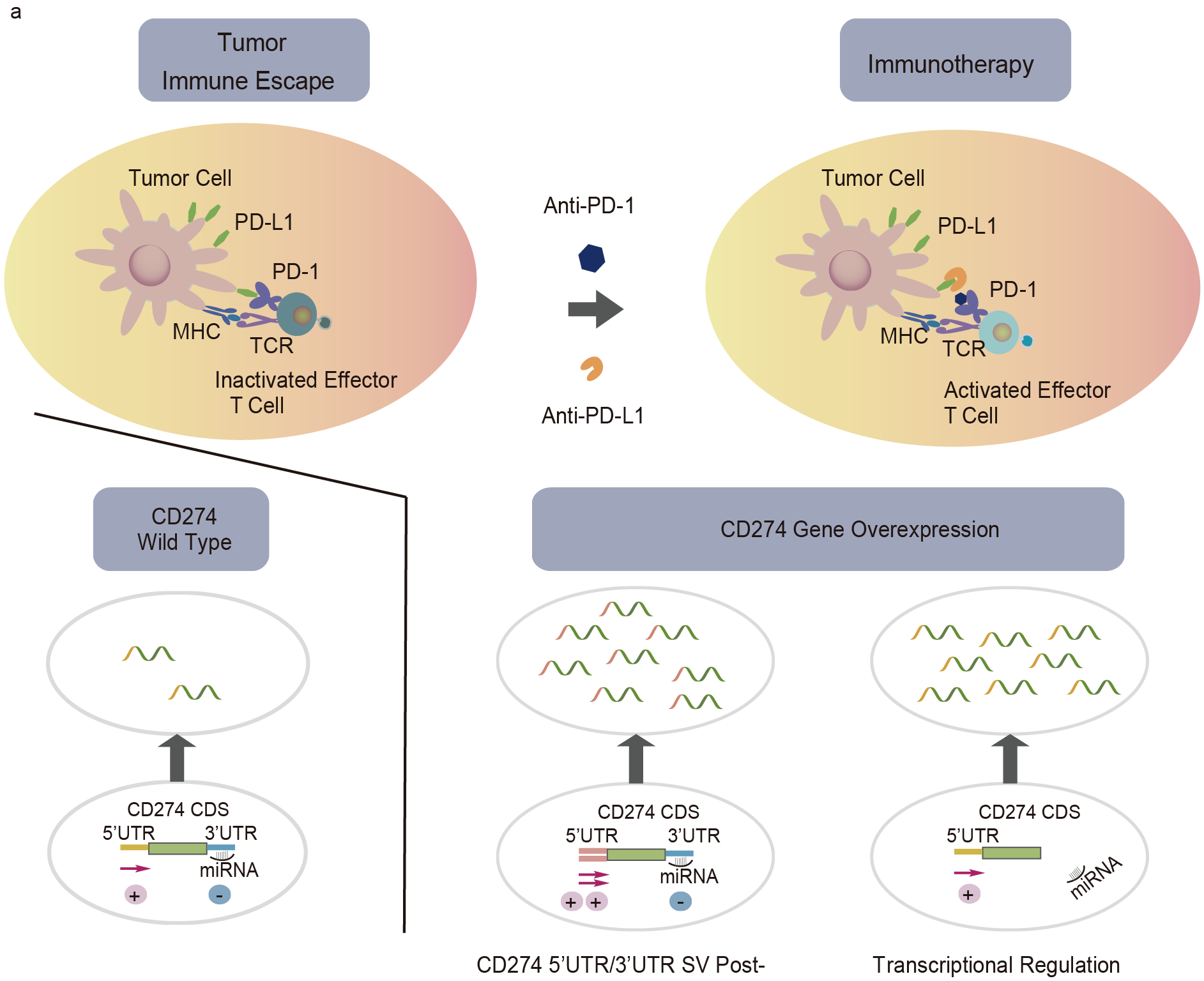

Supplement: Supplementary file 6 — Additional File 6 [file 41420_2023_1316_MOESM6_ESM.png]
