# Supplementary material for: Non-invasive plasma testing for CD274 UTR structural variations by next-generation sequencing in cancer
Source: Cell Death Discov. 2023 Jan 30;9:35. doi: 10.1038/s41420-023-01316-1 (PMC9887064; doi:10.1038/s41420-023-01316-1)
Supplement: Supplementary file 7 — Additional File 7 [file 41420_2023_1316_MOESM7_ESM.docx]

**Supplementary Table 1.** Concurrent alterations identified in patients.

| **Case** | **Mut Type** | **Gene** | **Exon** | | | **cds** | **Protein change** | | | | **Depth** | **Ratio** |
| --- | --- | --- | --- | --- | --- | --- | --- | --- | --- | --- | --- | --- |
| Case3 | SNV Indel | *TP53BP1*  (NM_001141980) | 14 | | | c.2839A>G | p.I947V | | | | 94/821 | 11.45% |
|  |  | *BIRC3*  (NM_182962) | 8 | | | c.1408A>G | p.T470A | | | | 97/1161 | 8.35% |
|  |  | *TAF1*  (NM_004606) | 23 | | | c.3605G>A | p.R1202H | | | | 125/1774 | 7.05% |
|  | CNV | Negative | | | | | | | | | | |
|  | TMB | 1.88Muts/Mb | | | | | | | | | | |
|  | TNB | 0.63/Mb | | | | | | | | | | |
|  | MSI | MSS | | | | | | | | | | |
|  | PD-L1  (IHC) | TPS : 60% | | | | | | | | | | |
|  |  | CPS : 65 | | | | | | | | | | |
| Case4 | SNV Indel | *KITLG*  (NM_000899) | 9 | | | c.800A>C | | p.E267A | | 210/4315 | | 4.87% |
|  |  | *TP53*  (NM_000546) | 7 | | | c.709A>G | | p.M237V | | 626/6404 | | 9.78% |
|  |  | *TSC2*  (NM_000548) | 14 | | | c.1443G>C | | p.E481D | | 315/2997 | | 10.51% |
|  | CNV | Negative | | | | | | | | | | |
|  | TMB | 4.38Muts/Mb | | | | | | | | | | |
|  | MSI | MSS | | | | | | | | | | |
|  | PD-L1  (IHC) | TPS:60% | | | | | | | | | | |
|  |  | CPS:65 | | | | | | | | | | |
| Case6 | SNV | *ALK*  (NM_004304) | 21 | | c.3436C>A | | | p.Q1146K | | 746/9339 | | 7.99% |
|  |  | *ATP11B*  (NM_014616) | 12 | | c.1067C>T | | | p.S356L | | 163/1886 | | 8.64% |
|  |  | *ATR*  (NM_001184) | 10 | | c.2101G>C | | | p.D701H | | 280/3458 | | 8.1% |
|  |  |  | 43 | | c.668G>C | | | p.R223T | | 293/310 | | 9.44% |
|  |  | *BAP1*  (NM_004656) | 9 | | c.709C>T | | | p.R237C | | 25/3872 | | 0.65% |
|  |  | *BRIP1*  (NM_032043) | 20 | | c.3376G>A | | | p.E1126K | | 258/4986 | | 5.17% |
|  |  |  | 5 | | c.474G>C | | | p.K158N | | 826/5251 | | 15.73% |
|  |  | *CD22*  (NM_001771) | 3 | | c.321C>A | | | p.H107Q | | 834/8058 | | 10.35% |
|  |  | *CD48*  (NM_001256030) | 1 | | c.23C>T | | | p.S8L | | 79/5104 | | 1.55% |
|  |  | *CDK12*  (NM_016507) | 1 | | c.385G>A | | | p.E129K | | 486/33984 | | 1.43% |
|  |  |  |  |  | c.403G>T | | | p.E135* | | 468/32426 | | 1.44% |
|  |  |  |  |  | c.559G>C | | | p.E187Q | | 474/30218 | | 1.57% |
|  |  |  |  |  | c.848C>A | | | p.S283* | | 486/26479 | | 1.84% |
|  |  |  |  |  | c.1926G>A | | | p.M642I | | 880/27479 | | 3.2% |
|  |  | *COP1*  (NM_001001740) | 1 | | c.224C>T | | | p.S75L | | 362/1175 | | 30.81% |
|  |  | *CSF1R*  (NM_005211) | 17 | | c.2308G>A | | | p.A770T | | 490/4542 | | 10.79% |
|  |  | *CSF3R*  (NM_000760) | 14 | | c.1819G>A | | | p.G607R | | 715/4865 | | 14.7% |
|  |  | *CTNNA1*  (NM_001290307) | 19 | | c.2525G>T | | | p.*842Lext*7 | | 370/3747 | | 9.87% |
|  |  | *CTNNA1*  (NM_001903) | 18 | | c.2475G>C | | | p.L825F | | 440/4364 | | 10.08% |
|  |  |  |  |  | c.2615G>C | | | p.R872T | | 522/5247 | | 9.95% |
|  |  |  |  |  | c.2629G>A | | | p.E877K | | 565/5295 | | 10.67% |
|  |  | *CUX1*  (NM_001202544) | 18 | | c.1648G>A | | | p.D550N | | 32/5395 | | 0.59% |
|  |  | *DAXX*  NM_001141970 | 6 | | c.1727C>T | | | p.S576F | | 666/5163 | | 12.9% |
|  |  | *DICER1*  (NM_030621) | 25 | | c.4886C>T | | | p.S1629L | | 458/5178 | | 8.85% |
|  |  |  |  |  | c.4874C>T | | | p.S1625F | | 457/5253 | | 8.7% |
|  |  | *ECT2L*  (NM_001077706) | 12 | | c.1334C>G | | | p.S445C | | 50/4410 | | 1.13% |
|  |  | *EP300*  (NM_001429) | 3 | | c.738G>T | | | p.M246I | | 23/1573 | | 1.46% |
|  |  | *EPCAM*  (NM_002354) | 6 | | c.559G>C | | | p.E187Q | | 123/2245 | | 5.48% |
|  |  | *ERBB2*  (NM_004448) | 4 | | c.466C>T | | | p.Q156* | | 1033/36363 | | 2.84% |
|  |  | *FANCD2*  (NM_001018115) | 11 | | c.859C>T | | | p.H287Y | | 476/4849 | | 9.82% |
|  |  | *FANCM*  (NM_020937) | 14 | | c.3801G>T | | | p.K1267N | | 211/3969 | | 5.32% |
|  |  | *GRIN2A*  (NM_001134407) | 13 | | c.4069C>G | | | p.L1357V | | 497/4814 | | 10.32% |
|  |  | *ICOSLG*  (NM_015259) | 5 | | c.716G>C | | | p.R239T | | 438/3500 | | 12.51% |
|  |  | *IKZF3*  (NM_012481) | 7 | | c.755G>C | | | p.R252T | | 459/29037 | | 1.58% |
|  |  | *INPPL1*  (NM_001567) | 21 | | c.2402G>T | | | p.R801L | | 247/2266 | | 10.9% |
|  |  | *JAK2*  (NM_004972) | 7 | | c.889C>T | | | p.Q297* | | 448/3038 | | 14.75% |
|  |  | *KMT2B*  (NM_014727) | 16 | | c.4123G>C | | | p.D1375H | | 312/3427 | | 9.1% |
|  |  |  | 18 | | c.4469G>A | | | p.G1490E | | 448/6964 | | 6.43% |
|  |  | *KMT2D*  (NM_003482) | 39 | | c.12188G>T | | | p.G4063V | | 635/5787 | | 10.97% |
|  |  | *MUC16*  (NM_024690) | 78 | | c.42852G>C | | | p.K14284N | | 771/6439 | | 11.97% |
|  |  |  | 4 | | c.31357C>G | | | p.Q10453E | | 522/5633 | | 9.27% |
|  |  |  | 3 | | c.21488T>A | | | p.L7163Q | | 676/7366 | | 9.18% |
|  |  |  |  |  | c.10803G>C | | | p.L3601F | | 551/4975 | | 11.08% |
|  |  |  | 1 | | c.7219C>G | | | p.L2407V | | 1843/6312 | | 29.2% |
|  |  | *MYCN*  (NM_005378) | 3 | | c.1327G>A | | | 419/6110 | | p.E443K | | 6.86% |
|  |  | *NF1*  (NM_000267) | 9 | | c.1016A>C | | | p.N339T | | 432/4012 | | 10.77% |
|  |  | *NOTCH2*  (NM_024408) | 34 | | c.6046C>T | | | p.L2016F | | 371/4223 | | 8.79% |
|  |  | *NOTCH4*  (NM_004557) | 23 | | c.4147G>A | | | p.V1383M | | 474/4002 | | 11.84% |
|  |  | *NSD3*  (NM_023034) | 23 | | c.3894G>C | | | p.E1298D | | 310/2297 | | 13.5% |
|  |  | *PARP2*  (NM_005484) | 8 | | c.646G>A | | | p.E216K | | 234/1992 | | 11.75% |
|  |  | *PARP3*  (NM_001003931) | 4 | | c.354G>C | | | p.K118N | | 458/3670 | | 12.48% |
|  |  | *PIK3CA*  (NM_006218) | 2 | | c.223C>G | | | p.Q75E | | 815/7766 | | 10.49% |
|  |  | *PTPRT*  (NM_133170) | 23 | | c.3064G>C | | | p.D1022H | | 1159/6064 | | 19.11% |
|  |  | *RAD50*  (NM_005732) | 14 | | c.2394C>G | | | p.F798L | | 356/2741 | | 12.99% |
|  |  |  | 16 | | c.2708G>C | | | p.R903T | | 144/1076 | | 13.38% |
|  |  | *RAF1*  (NM_002880) | 2 | | c.115C>T | | | p.Q39* | | 529/6448 | | 8.2% |
|  |  | *RECQL4*  (NM_004260) | 5 | | c.1120C>T | | | p.L374F | | 380/3445 | | 11.03% |
|  |  | *RHOA*  (NM_001664) | 2 | | c.50G>A | | | p.G17E | | 569/5538 | | 10.27% |
|  |  | *RUNX1*  (NM_001754) | 9 | | c.1366G>A | | | p.E456K | | 272/2888 | | 9.42% |
|  |  | *SAMHD1*  (NM_015474) | 15 | | c.1651C>T | | | p.R551* | | 234/3044 | | 7.69% |
|  |  | *SPEN*  (NM_015001) | 2 | | c.92G>A | | | p.R31H | | 407/2970 | | 13.7% |
|  |  | *SPOP*  (NM_001007228) | 3 | | c.96C>G | | | p.F32L | | 308/20180 | | 1.53% |
|  |  | *SPRED1*  (NM_152594) | 4 | | c.389C>G | | | p.S130* | | 157/1729 | | 9.08% |
|  |  | *TERT*  (NM_198253) | 2 | | c.1566G>C | | | p.R522S | | 45/7319 | | 0.61% |
|  |  | *TP53*  (NM_000546) | 6 | | c.584T>C | | | p.I195T | | 1725/6381 | | 27.03% |
|  |  | *TP53BP1*  (NM_001141980) | 12 | | c.2494G>C | | | p.E832Q | | 501/3927 | | 12.76% |
|  |  | *TYK2*  (NM_003331) | 12 | | c.1681C>G | | | p.L561V | | 428/5454 | | 7.85% |
|  |  | *USP9X*  (NM_001039590) | 29 | | c.4253G>T | | | p.G1418V | | 101/1215 | | 8.31% |
|  |  |  | 36 | | c.6136C>T | | | p.Q2046* | | 264/2999 | | 8.8% |
|  |  | *ZFHX3*  (NM_006885) | 9 | | c.6445C>T | | | p.R2149C | | 1770/7103 | | 24.92% |
|  |  |  |  |  | c.5800C>G | | | p.P1934A | | 551/7205 | | 7.65% |
|  | Indel | *CDK12*  (NM_016507) | 1 | | c.841_842del  insAC | | | p.E281T | | 357/25135 | | 1.42% |
|  |  | *MKNK1*  (NM_003684) | 13 | | c.1143_1144  delinsTT | | | p.K381_G3  82delinsN* | | 501/4549 | | 11.01% |
|  |  | *NTRK3*  (NM_001012338) | 14 | | c.1424_1425  delinsGT | | | p.E475G | | 429/5905 | | 7.27% |
|  |  | *SMC3*  (NM_005445) | 13 | | c.1144_1145  delinsCT | | | p.G382L | | 279/3621 | | 7.71% |
|  | CNV | **Gene** | | **Mut Type** | | | | | **Copy Number** | | | |
|  |  | *CCND1*  (NM_053056) | | Copy number increase | | | | | 5.36 | | | |
|  |  | *ERBB2*  (NM_004448) | | Copy number increase | | | | | 10.51 | | | |
|  |  | *FGF19*  (NM_005117) | | Copy number increase | | | | | 5.71 | | | |
|  |  | *FGF3*  (NM_005247) | | Copy number increase | | | | | 5.45 | | | |
|  |  | *FGF4*  (NM_002007) | | Copy number increase | | | | | 4.25 | | | |
|  |  | *GNA13*  (NM_006572) | | Copy number increase | | | | | 3.19 | | | |
|  |  | *IGF1R*  (NM_000875) | | Copy number increase | | | | | 2.96 | | | |
|  |  | *PPM1D*  (NM_003620) | | Copy number increase | | | | | 3.44 | | | |
|  | TMB | 1.88Muts/Mb | | | | | | | | | | |
|  | TNB | 10.0/Mb | | | | | | | | | | |
|  | MSI | MSS | | | | | | | | | | |
|  | PD-L1  (IHC) | TPS:60% | | | | | | | | | | |
|  |  | CPS:65 | | | | | | | | | | |
| Case8 | **Mut Type** | **Gene** | **Exon** | | | **cds** | **Protein change** | | | | **Ratio** | |
|  | SNV | *BCORL1*  (NM_021946) | 9 | | | c.4556C>T | p.T1519I | | | | 5.61% | |
|  |  | *HIST1H1E*  (NM_005321) | 1 | | | c.494C>T | p.A165V | | | | 12.93% | |
|  |  | *IKZF3*  (NM_012481) | 8 | | | c.1033G>A | p.A345T | | | | 9.89% | |
|  |  | *IRF8*  (NM_002163) | 2 | | | c.62G>A | p.S21N | | | | 3.61% | |
|  |  | *SOCS1*  (NM_003745) | 2 | | | c.457C>T | p.H153Y | | | | 3.23% | |
|  |  |  |  |  |  | c.429C>G | p.S143R | | | | 4.51% | |
|  | Indel | *KLF*  *(NM_06270)* | 2 | | | c.857_868del12 | p.S286_L289delSSHL | | | | 7.63% | |
|  |  | *SOCS1*  *(NM_003745)* | 2 | | | c.541_551delinsTG | p.R181_A184delinsC | | | | 10.97% | |
|  |  |  |  |  |  | c.4_15delinsATAG | p.V2Ifs*112 | | | | 9.06% | |
|  | CNV | Negative | | | | | | | | | | |
|  | TMB | 5.77Muts/Mb | | | | | | | | | | |
|  | MSI | MSS | | | | | | | | | | |
|  | PD-L1  (IHC) | TPS : 30% | | | | | | | | | | |
|  |  | CPS : 30 | | | | | | | | | | |
