# Supplementary material for: Non-invasive plasma testing for CD274 UTR structural variations by next-generation sequencing in cancer
Source: Cell Death Discov. 2023 Jan 30;9:35. doi: 10.1038/s41420-023-01316-1 (PMC9887064; doi:10.1038/s41420-023-01316-1)
Supplement: Supplementary file 8 — Additional File 8 [file 41420_2023_1316_MOESM8_ESM.docx]

**Supplementary Table 2. mIF results.**

| Case | Tissue Category | #TOTAL | #CD68 (POS) | %CD68 (POS) | #CD8 (POS) | %CD8 (POS) | #FoxP3 (POS) | %FoxP3 (POS) | #PanCK (POS) | %PanCK (POS) | #PD-1 (POS) | %PD-1 (POS) | #PD-L1 (POS) | %PD-L1 (POS) | %PD-L1 (POS) / %CD68 (POS) | %PD-L1 (POS) / %CD8 (POS) |
| --- | --- | --- | --- | --- | --- | --- | --- | --- | --- | --- | --- | --- | --- | --- | --- | --- |
|  |  |  |  |  |  |  |  |  |  |  |  |  |  |  |  |  |
| Case1 | Tumor | 847 | 8 | 0.009 | 6 | 0.007 | 5 | 0.006 | 785 | 0.927 | 0 | 0 | 549 | 0.648 | 68.625 | 91.5 |
| Case2 | Tumor | 2020 | 129 | 0.064 | 97 | 0.048 | 74 | 0.037 | 1707 | 0.845 | 515 | 0.255 | 1848 | 0.915 | 14.326 | 19.052 |
| Case3 | Tumor | 1299 | 22 | 0.017 | 99 | 0.076 | 58 | 0.045 | 1238 | 0.953 | 1 | 0.001 | 1200 | 0.924 | 54.545 | 12.121 |
| Case4 | Tumor | 671 | 198 | 0.295082 | 238 | 0.354694 | 29 | 0.043219 | 264 | 0.393443 | 156 | 0.232489 | 421 | 0.627422 | 2.126263 | 1.768908 |
| Case7 | Tumor | 3597 | 1523 | 0.423 | 942 | 0.262 | 181 | 0.05 | 1 | 0 | 2182 | 0.607 | 3319 | 0.923 | 2.179 | 3.523 |
| Case8 | Tumor | 3319 | 824 | 0.248268 | 837 | 0.252184 | 757 | 0.228081 | 139 | 0.04188 | 3098 | 0.933414 | 2418 | 0.728533 | 2.934466 | 2.888889 |
